# Metallic Electrooptic Effect in Twisted Double-Bilayer Graphene

**DOI:** 10.1021/acs.nanolett.5c05662

**Published:** 2026-05-28

**Authors:** D. J. P. de Sousa, N. Roldan-Levchenko, C. O. Ascencio, J. D. S. Forte, Paul M. Haney, Tony Low

**Affiliations:** † Department of Electrical and Computer Engineering, University of Minnesota, Minneapolis, Minnesota 55455, United States; ‡ School of Physics and Astronomy, University of Minnesota, Minneapolis, Minnesota 55455, United States; § Physical Measurement Laboratory, 10833 National Institute of Standards and Technology, Gaithersburg, Maryland 20899-6202, United States; ∥ Department of Physics, University of Minnesota, Minneapolis, Minnesota 55455, United States

**Keywords:** Twisted double-bilayer graphene, magnetoelectric electro-optic effect, Berry curvature, orbital magnetic moment, moiré materials, terahertz circular dichroism

## Abstract

Recent theoretical advances have highlighted the role of Bloch state intrinsic properties in enabling unconventional electro-optic (EO) phenomena in bulk metals, offering novel strategies for dynamic optical control in quantum materials. Here, we identify an alternative EO mechanism in bulk metallic systems that arises from the interplay between Berry curvature and the orbital magnetic moment of Bloch electrons. Focusing on twisted double-bilayer graphene (TDBG), we show that the enhanced intrinsic properties of moiré Bloch bands give rise to a sizable linear magnetoelectric EO response, a first-order, electric-field-induced non-Hermitian correction to the gyrotropic magnetic susceptibility. This mechanism dominates in *C*_3*z*_-symmetric TDBG, where EO contributions originating from the Berry curvature dipole (BCD) are symmetry-forbidden. Our calculations reveal giant, gate-tunable linear and circular dichroism in the terahertz regime, establishing a robust and tunable platform for ultrafast EO modulation in two-dimensional materials beyond the BCD paradigm.

## Graphical abstract

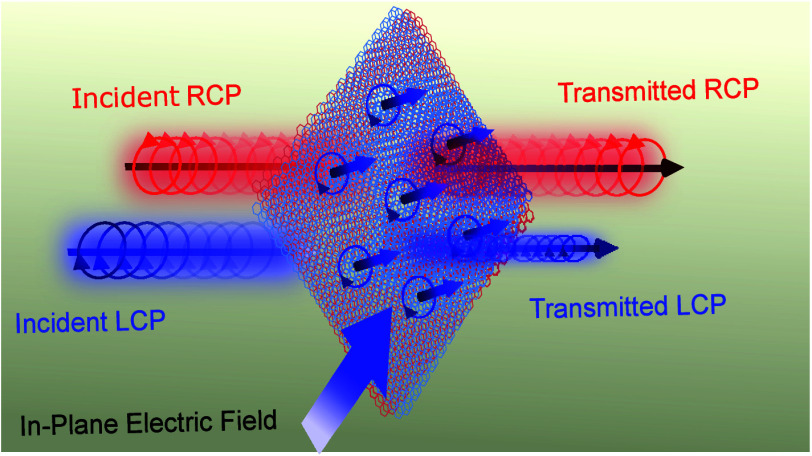

Noncentrosymmetric metals have recently emerged as a fertile platform for unconventional electro-optic (EO) phenomena, revealing rich low-frequency responses previously thought to be suppressed in bulk metallic systems.^1–6^ In such materials, the intrinsic properties of Bloch states on the Fermi surface, such as the Berry curvature and the orbital magnetic moment, can couple static and optical fields in a fundamental manner, giving rise to nonreciprocal effects in the linear optical regime.^3,6^ While previous studies have primarily focused on EO effect mediated by the Berry curvature dipole (BCD),^1,3,4^ the first moment of the Berry curvature in momentum space,^7–11^ new theoretical developments have revealed that the orbital magnetic moment texture of Bloch electrons on the Fermi surface can generate an entirely distinct class of EO responses, of which the so-called magnetoelectric EO effects is present in time-reversal symmetric systems.^6^ However, the effects remain largely unexplored in realistic material platforms, and their role in enabling optical control have yet to be demonstrated or quantified.

In this work, we identify twisted double bilayer graphene (TDBG) as an ideal platform for realizing giant, gate-tunable, magnetoelectric EO effects in the terahertz range. We show that the presence of *C*_3*z*_ symmetry in TDBG enables leading-order linear magnetoelectric EO response, with moiré Bloch states enabling bias-induced magnetoelectric coefficients exceeding 20000 μ_*B*_/V·nm, with a strong dependence on twist angle and vertical displacement field. The resulting circular dichroism (CD) exhibits a distinct angular dependence compared to previous studies in twisted systems,^12–14^ vanishing at normal incidence. While CD in unbiased moiré systems has been attributed to in-plane magnetic moments,^13^ as illustrated in Figure 1(a), the linear magnetoelectric EO response explored here generates net out-of-plane moments [Figure 1(b)], leading to qualitatively different CD signatures. These findings position TDBG as a highly tunable platform for probing Fermi-surface orbital magnetization via optical means and provide a concrete material realization of metallic EO control beyond the conventional Berry curvature dipole (BCD) paradigm.

**Figure 1:**
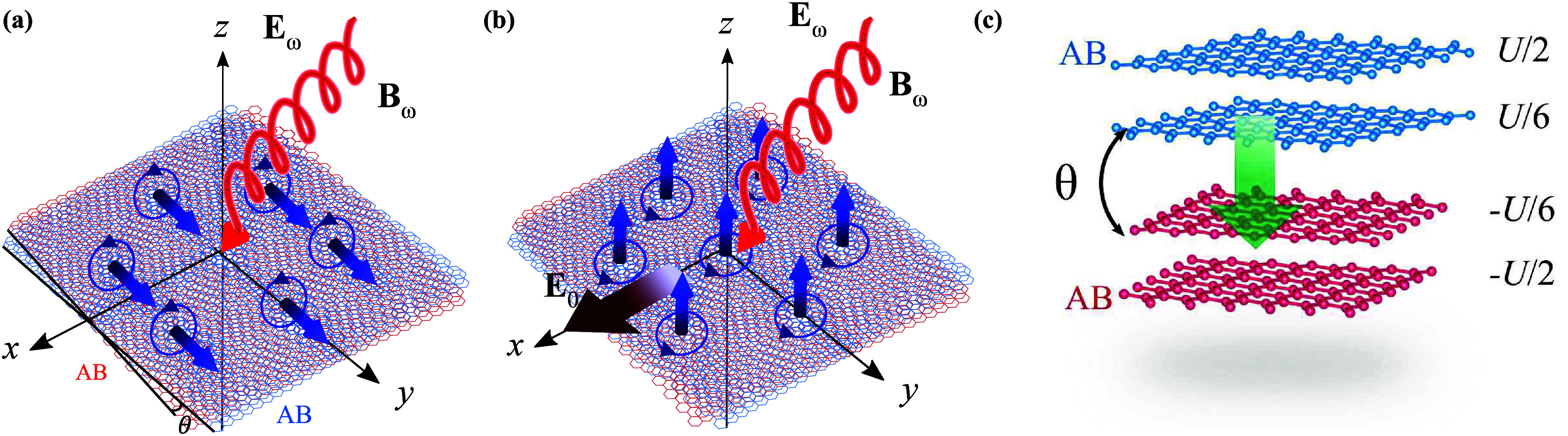
(a) In bias-free twisted graphene systems, circular dichroism (CD) has been attributed to the interaction between in-plane magnetic moments **M** and the optical fields **E**_ω_, **B**_ω_. These in-plane moments arise from chiral interlayer moiré coupling at finite twist angles θ. (b) In contrast, the metallic magnetoelectric electro-optic effect generates out-of-plane magnetic moments in twisted graphene under static electric fields **E**_0_, which couple with the optical fields to produce a distinct dichroic response. (c) Schematic setup for modeling the vertical displacement field in twisted double bilayer graphene. Following ref,^15^ we neglect screening effects and assume a vertical bias difference of *U*/3 between adjacent graphene layers.

In the low-frequency limit, *ℏω* ≪ϵ_gap_, with ϵ_gap_ being the optical gap, the system’s electromagnetic response is dominated by intraband transitions. Within the dilute impurity limit, γ ≪ ω, where τ = 1/γ is the relaxation time, the semiclassical approach adopted here confines the frequency range to γ ≪ ω ≪ϵ_gap_/*ℏ*.

The constitutive relation for inversion-broken and time-reversal symmetric systems takes the form

1 $$J_{0}^{\beta }\left(\omega \right)=\sigma_{Drude}^{\alpha \beta }\left(\omega \right)E_{\omega }^{\beta }+\sigma_{GME}^{\alpha \beta }\left(\omega \right)B_{\omega }^{\beta }$$

capturing the conventional AC Drude response and the gyrotropic magnetic effect (GME) derived from the magnetic moment texture of Bloch electrons on the Fermi surface.^16,17^

The presence of a static electric field, **E**_0_, alters the system’s electromagnetic response by introducing corrections to the constitutive relation, leading to electro-optical (EO) effects:^18^ $J_{0}^{\beta }\left(\omega \right)\to J_{0}^{\beta }\left(\omega \right)+J_{EO}^{\beta }$ , where $J_{EO}^{\beta }\left(\omega \right)$ can be written most generally as

2 $$J_{EO}^{\alpha }\left(\omega \right)=\sigma_{D}^{\alpha \beta }\left(\omega \right)E_{\omega }^{\beta }+\sigma_{G}^{\alpha \beta }\left(\omega \right)B_{\omega }^{\beta }$$

Together, the tensors **σ**_Drude_(ω), **σ**_GME_(ω), **σ**_D_(ω) and **σ**_G_(ω), account for the relevant optical responses arising from the coupling between optical fields, static bias and the wave function of Bloch electrons in inversion-broken, time-reversal symmetric systems. The nature of these responses has been addressed in previous works.^1,3,18^ They are summarized as

3 $$\sigma_{Drude}^{\alpha \beta }\left(\omega \right)=\frac{e^{2}}{\gamma -i\omega }V^{\alpha \beta },\sigma_{GME}^{\alpha \beta }\left(\omega \right)=e\frac{i\omega }{i\omega -\gamma }K^{\alpha \beta }$$

with $V^{\alpha \beta }=\underset{nk}{\sum }\left(-\partial f_{nk}^{0}/\partial \epsilon_{nk}\right)v_{nk}^{\alpha }v_{nk}^{\beta }$ and $K^{\alpha \beta }=\underset{nk}{\sum }\left(-\partial f_{nk}^{0}/\partial \epsilon_{nk}\right)v_{nk}^{\alpha }m_{nk}^{\beta }$ , describing unbiased intraband optical responses. The next contribution, **σ**_D_(ω), is typically written as a sum of two responses,^1,18^ such that

4 $$\sigma_{D}^{\alpha \beta }\left(\omega \right)=-\frac{e^{3}}{\hbar }\underset{\kappa \lambda }{\sum }\left[\frac{1}{\gamma -i\omega }\epsilon_{\alpha \kappa \lambda }D^{\kappa \beta }E_{0}^{\lambda }-\frac{1}{\gamma }\epsilon_{\alpha \beta \lambda }D^{\lambda \kappa }E_{0}^{\kappa }\right]$$

where the summation is most generally over κ, λ = *x*, *y*, *z*, with $D^{\alpha \beta }=\underset{nk}{\sum }\left(-\partial f_{nk}^{0}/\partial \epsilon_{nk}\right)\Omega_{nk}^{\alpha }v_{nk}^{\beta }$ , capturing the contributions derived from the BCD (up to a *ℏ* factor),^7,19^ and

5 $$\sigma_{G}^{\alpha \beta }\left(\omega \right)=-\frac{e^{2}}{\hbar }\frac{i\omega }{i\omega -\gamma }\underset{\kappa \lambda }{\sum }\epsilon_{\alpha \kappa \lambda }G^{\kappa \beta }E_{0}^{\lambda }$$

with $G^{\alpha \beta }=\underset{nk}{\sum }\left(-\partial f_{nk}^{0}/\partial \epsilon_{nk}\right)\Omega_{nk}^{\alpha }m_{nk}^{\beta }$ , describing the bias-induced correction to the GME (i.e., magnetoelectric EO effect^18^). In this work, we will also refer to the quantity $\chi_{nk}^{\alpha \beta }=\Omega_{nk}^{\alpha }m_{nk}^{\beta }$ describing the local magnitude of this effect within the Brillouin zone. Here, **v**_*n* **k**_ = (1/*ℏ*)∇_**k**_ϵ_*n* **k**_ is the Bloch velocity, **Ω**_*n* **k**_ is the Berry curvature and **m**_*n* **k**_ is the magnetic moment arising from spin and the self-rotation of Bloch wave packets.^20^

Unlike previous works that focused solely on the contribution from $\sigma_{D}^{\alpha \beta }\left(\omega \right)$ ,^1,3,4^ this study underscores the critical role of the bias-induced magnetoelectric term, $\sigma_{G}^{\alpha \beta }\left(\omega \right)$ , offering a more complete picture of metallic EO responses. In this work, we investigate TDBG^15,21–25^ as a promising platform for metallic EO effects. Remarkably, we demonstrate that these systems exhibit *leading-order* giant magnetoelectric EO responses due to their point group symmetries, which enforce **D** = **0** and **K** = **0** in the idealized *C*_3*z*_-symmetric limit. In realistic devices, symmetry breaking due to nonuniform strain distributions may allow for the existence of GME and BCD-induced responses, with potentially comparable magnitudes to the effect predicted here. Nevertheless, we demonstrate that the magnetoelectric EO effect produces a unique dependence of ellipticity on the incidence angle. This distinct angular signature is not shared by GME or BCD contributions, providing a clear experimental pathway to isolate and probe the predicted effect. In the following, we discuss the symmetry-enforced form of the metallic EO response tensors.

Before addressing the electronic structure and the optical responses induced by a static electric field in this system, we first focus on the symmetry-dictated form of the response tensors.

The point group of twisted (double) bilayer graphene, which imposes constraints on the components of the magnetoelectric response tensors, depends on the type of stacking and twist angle.^26–29^ The point group associated with the TDBG system considered here is *D*_3_,^30^ which is generated by a 3-fold axis along the stacking direction, *C*_3*z*_, and an in-plane 2-fold axis, *C*_2*y*_. We will now argue that the presence of a *C*_3*z*_ symmetry alone is sufficient to enforce the vanishing of the BCD pseudovector components, *D* ^*zx*^ and *D* ^*zy*^, as well as *K* ^*zx*^ and *K* ^*zy*^ capturing the GME.

*C*_3*z*_ is a proper rotation, so the Berry curvature, magnetic moment and velocity components transform in an identical manner. Accounting for this symmetry and integrating over the BZ gives $D_{n}^{zx}=-\frac{\sqrt{3}}{3}D_{n}^{zy}$ and $D_{n}^{zy}=\frac{\sqrt{3}}{3}D_{n}^{zx}$ , $K_{n}^{zx}=-\frac{\sqrt{3}}{3}K_{n}^{zy}$ and $K_{n}^{zy}=\frac{\sqrt{3}}{3}K_{n}^{zx}$ .^27^ Thus, $D_{n}^{zx}=D_{n}^{zy}=0$ , as well as $K_{n}^{zx}=K_{n}^{zy}=0$ . The component-wise symmetry analysis performed in ref 27 directly implies in $D_{n}^{zx}=D_{n}^{zy}=0$ . We have systematically performed a symmetry analysis on all graphene systems of interest beginning with the monolayer case and building up to TDBG in levels of decreasing symmetry (please see Supporting Information^27^). Our analysis shows that all cases must have vanishing $D_{n}^{zx}$ and $D_{n}^{zy}$ ( $K_{n}^{zx}$ and $K_{n}^{zy}$ ) by virtue of *C*_3*z*_. Our conclusions are fully consistent with the point group analysis of ref^7^ applied to the components of the BCD.

As we have shown, nonzero *D* ^*zx*^, *D* ^*zx*^ (*K* ^*zx*^, *K* ^*zy*^), require *C*_3*z*_ symmetry breaking, commonly achieved through strain.^31,32^ However, in moiré devices the strain is typically nonuniform, such that the net BCD (and likewise the GME) is expected to be strongly suppressed when averaged over the typical micrometer-scale spot size of conventional terahertz lasers. Consequently, we expect that our results, which are based on a *C*_3*z*_-symmetric system, provide a reasonable and physically relevant description of the experimental setup proposed in this work. Hence, in the absence of strain, the nonlinear magnetoelectric electro-optical effect discussed here is the leading order magnetoelectric response. It is important to note that, linear GME $\left(\propto E_{\omega }^{\alpha }\right)$ is not possible within the TDBG point group, but the nonlinear $\left(\propto E_{0}^{\gamma }E_{\omega }^{\alpha }\right)$ counterpart is. In the following, we study the electronic structure properties enabling sizable metallic magnetoelectric EO effects in this system.

We begin by focusing on the electronic structure of TDBG, highlighting quantities relevant to the magnetoelectric EO effect. We adopt the Bistritzer-MacDonald continuum (BM) model to address the electronic structure of TDBG.^15,27,33^ The electrostatic potential due to a vertical displacement field is modeled as a linear potential drop of magnitude *U*, as shown in Figure 1(c), where screening and many-body effects have been neglected.^27,35^ For simplicity, we explicitly focus on the *K*-valley BM model, noting that the corresponding results for the *K*′-valley can be obtained via time-reversal symmetry. We confine our description to small deviations off the 1.75° configuration, to ensure that our single-particle model is an accurate description: While previous works have reported correlated phases for 0.8° < θ < 1.5° ^36,37^ and charge density wave states at larger angles θ ≈ 2.37°,^38^ attributed to the emergence of single-particle flat bands and bias-induced electron–hole band nesting, respectively, the electronic states near the θ ≈ 1.75° configuration are well described by the single-particle continuum model utilized here.^15^ In this regime, under small *U*, the bands are narrow but not flat, and the conditions for electron–hole nesting are not met. Our single-particle description is expected to be valid for θ > 1.6° and *U* < 80 meV. In fact, the electronic band structure of the 1.75° TDBG at a vanishing displacement field (*U* = 0 meV) is shown in Figure 2(a). The moiré interlayer coupling leads to narrow low-energy bands, with band widths of ≈50 meV for the top valence and bottom conduction states, separated by an energy gap of ≈20 meV.

**Figure 2:**
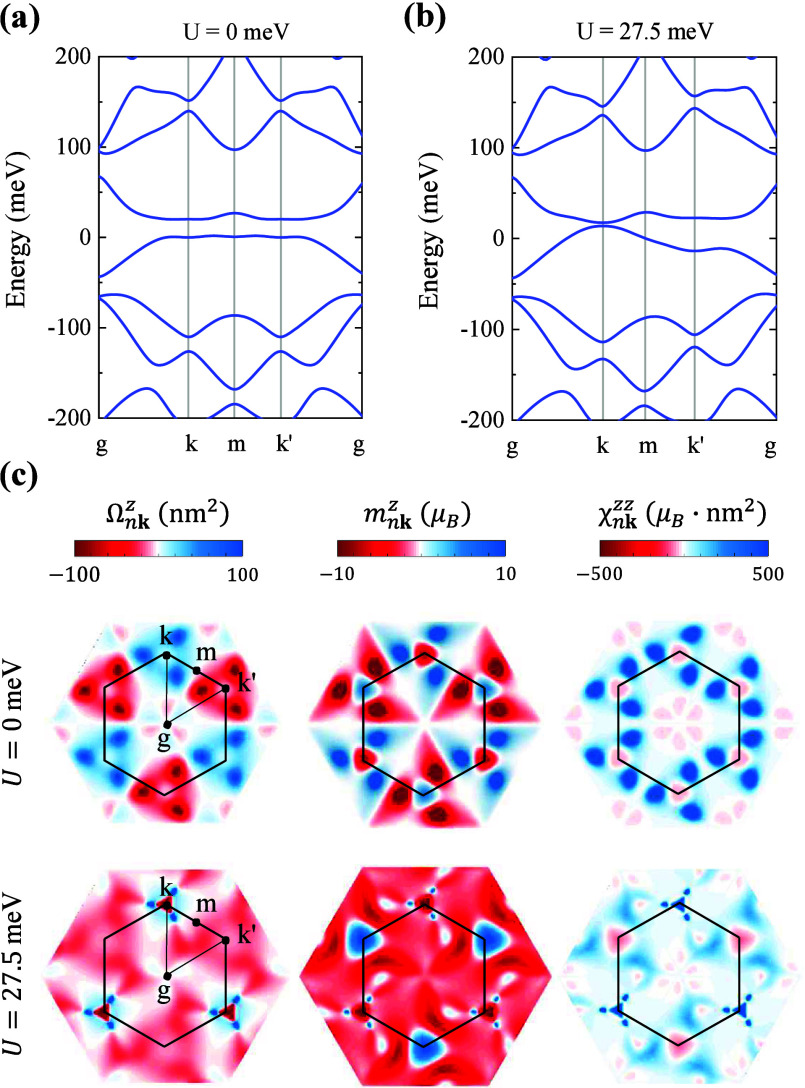
(a,b) Electronic structure of double-bilayer graphene twisted by θ = 1.75°, under vertical bias (a) *U* = 0 meV and (b) *U* = 27.5 meV for the K valley Bistritzer-MacDonald model. (c) Associated momentum-resolved Berry curvature, orbital magnetic moment and the bias-induced magnetoelectric coupling of Bloch electrons of the topmost valence states. Results for the K’ valley Bistritzer-MacDonald model are obtained by means of a time-reversal operation (i.e., by means of the prescription $\Omega_{nk}^{z}\left(K‐\mathrm{valley}\right)\to -\Omega_{nk}^{z}\left(K^{\prime }-\mathrm{valley}\right)$ , $m_{nk}^{z}\left(K‐\mathrm{valley}\right)\to -m_{nk}^{z}\left(K^{\prime }-\mathrm{valley}\right)$ and $\chi_{nk}^{zz}\left(K‐\mathrm{valley}\right)\to \chi_{nk}^{zz}\left(K^{\prime }-\mathrm{valley}\right)$ , where, $\chi_{nk}^{zz}=\Omega_{nk}^{z}m_{nk}^{z}$ ).

A finite vertical displacement field radically alters the electronic structure of TDBG.^24,25,30^ As the magnitude of the bias |*U*| increases, it drives a gap closure followed by a reopening at either the k or k’ valley of the moiré Brillouin zone, determined by the sign of *U*.^27^ Figure 2(b) displays the band structure in the vicinity of the gap-closing regime for *U* = 27.5 meV, where the gap at the k point is reduced to ≈3.7 meV, while it is enhanced to ≈33 meV at the k’ point. Further increase of *U* induces a gap reopening at the k point.

This high degree of tunability in the electronic structure via *U* has a direct influence on both the Berry curvature and the orbital magnetic moment of the Bloch states. Figure 2(c) presents the momentum-resolved maps of the out-of-plane components of the Berry curvature $\Omega_{nk}^{z}$ , orbital magnetic moment $m_{nk}^{z}$ , and their product $\chi_{nk}^{zz}=\Omega_{nk}^{z}m_{nk}^{z}$ (the magnetoelectric EO effect integrand), evaluated at midgap for *U* = 0 meV (top row) and *U* = 27.5 meV (bottom row). At zero bias, $\Omega_{nk}^{z}$ and $m_{nk}^{z}$ exhibit symmetric distributions in magnitude, with opposite signs centered around the k and k’ valleys. The presence of a finite displacement field (*U* = 27.5 meV), however, induces a marked asymmetry reflecting the bias-induced tendency for gap closure at k and enhancement at k’, as observed in the band structure. Consequently, $\chi_{nk}^{zz}$ becomes strongly localized in momentum space around the k valley, with a corresponding suppression near the k’ valley.

Recall that the results shown in Figure 2(c) correspond to the *K*-valley of each constituent bilayer graphene. The analogous quantities for the *K*′-valley are related by time-reversal symmetry, which imposes $\Omega_{nk}^{K}=-\Omega_{nk}^{K^{\prime }}$ and $m_{nk}^{K}=-m_{nk}^{K^{\prime }}$ .^39^ While both the Berry curvature and the orbital magnetic moment are odd under time-reversal, their product—the magnetoelectric response tensor **χ**_*n* **k**_ = **Ω**_*n* **k**_ **m**_*n* **k**_—is even. As a result, time-reversal symmetry prohibits the emergence of a net anomalous Hall current or total orbital magnetization, as expected, but permits nonvanishing magnetoelectric EO responses.

Given these salient features, we discuss now their impact on the intraband optical conductivity of TDBG in the presence of **E**_0_. The total metallic response of the system is dictated by the Drude conductivity **σ**_Drude_(ω) and the magnetoelectric EO conductivity **σ**_G_(ω). To quantify the Drude response, we define the frequency-independent quantity σ_*E*_ = *e* ^2^ *V*, where *V* is a diagonal element of the **V** tensor (because the system is isotropic *V* ^*xx*^ = *V* ^*yy*^ = *V*). Figure 3(a) shows σ_*E*_ obtained from the BM model for a few values of *U* near 27.5 meV, assuming γ = 10^–11^ rad/s (τ = 10 ps). In addition to magnitude, these results qualitatively reflect the behavior across the gap-closing and reopening regime, which will become important in upcoming analysis. To quantify the magnetoelectric EO response, we define the frequency-independent quantity α = (*e* ^2^/*γℏ*)*E*_0_ *G* ^*zz*^, where $G^{zz}=\underset{nk}{\sum }\left(-\partial f_{nk}^{0}/\partial \epsilon_{nk}\right)\chi_{nk}^{zz}$ , is the *zz* components of the magnetoelectric EO tensor. The quantity α is the effective magnetoelectric coupling coefficient induced by *E*_0_ = |**E**_0_|. Figure 3(b) presents the results for α obtained from the BM model for the same set of values of *U* near 27.5 meV. Following ref,^40^ we assume *E*_0_ = 10^4^ V/m. The results show that α peaks near the energy gap and changes sign between electron and hole bands.

**Figure 3:**
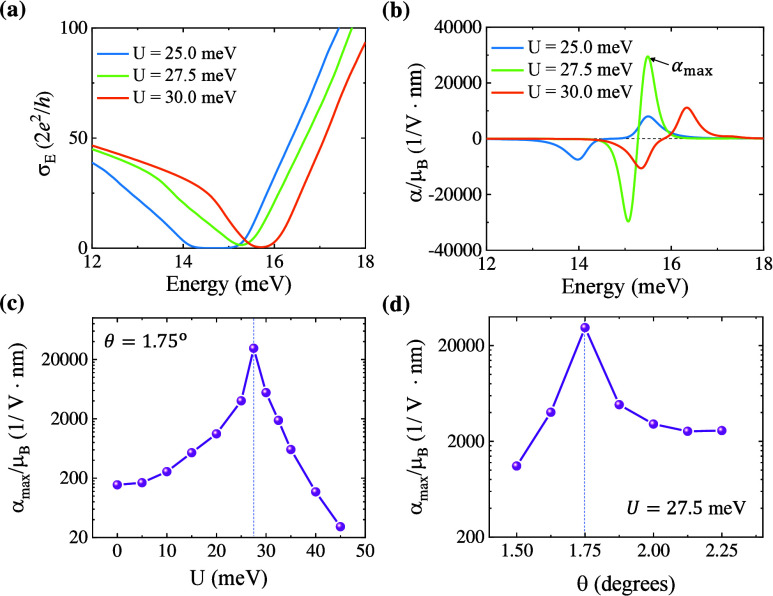
General metallic optical response of *C*_3*z*_ symmetric twisted double bilayer graphene, given in eq 6, depends on two parameters, σ_*E*_ and α, capturing the Drude conductivity and the bias induced metallic magnetoelectric EO response. (a) σ_*E*_ = *e* ^2^ *V* and (b) α as a function of the Fermi energy at θ = 1.75°, as obtained from the Bistritzer-MaDonald model. We have set, τ = 10 ps and $E_{0}^{y}=10^{4}$ V/m. Evolution of the maximum attainable bias-induced magnetoelectric coupdling α_max_ as a fuction of (c) vertical bias *U* and (d) twist angle θ.

The behavior of the BM model can be captured by an effective model describing states near the band gap at the k point, where a gapped Dirac Hamiltonian offers an accurate representation for the band structure.^27^ Within this framework, we obtain $G^{zz}=e{\left(\hbar v_{F}\Delta \right)}^{2}/32\pi \hbar \mu^{4}$ for the conduction band (μ > Δ/2) and $G^{zz}=-e{\left(\hbar v_{F}\Delta \right)}^{2}/32\pi \hbar \mu^{4}$ for the valence band (μ < – Δ/2), where Δ*i*s the bandgap, *v*_*F*_ is the Fermi velocity, and μ is the chemical potential. The pronounced 1/μ^4^ dependence accounts for the sharp peaks observed near the band edges. The model predicts that the maximum possible value occurs precisely at μ = ± Δ/2, with a magnitude scaling as $\left|G^{zz}\right|\propto {\left(\hbar v_{F}/\Delta \right)}^{2}$ . Therefore, a smaller gap leads to a larger α. This trend is clearly reflected in Figure 3(b), where the magnitude of α peaks at *U* = 27.5 meV, corresponding to the configuration nearest to gap closure. Note that the spectral broadening present in the numerical simulations displaces the peak in α slightly away from the band edge, resulting in the smoothened profile seen in the figure.

Remarkably, the maximum attainable bias-induced magnetoelectric coefficient, α_max_, reaches exceptionally large values as high as 20000 μ_*B*_/V·nm, where μ_*B*_ denotes the Bohr magneton, under a moderate external field of *E*_0_ = 10^4^ V/m. This value exceeds, by roughly an order of magnitude, the giant magnetoelectric response associated with the **K** tensor in strained twisted bilayer graphene, as reported in ref.^40^ The highly tunable electronic structure of TDBG translates into a remarkable degree of control over the magnetoelectric response. To illustrate this, the dependence of α_max_ on the vertical displacement field *U* and twist angle θ is shown in Figure 3(c) and (d). While α_max_ is strongly sensitive to *U*, it saturates around 2000 μ_*B*_/V·nm for twist angles θ > 2° at *U* = 27.5 meV. These results highlight TDBG as an exceptional and highly tunable platform for realizing metallic magnetoelectric EO effects. Next, we provide a practical analysis of how this effect manifests in optical dichroism, outlining a clear experimental route for its detection.

We examine how the giant metallic magnetoelectric EO effect gives rise to circular dichroism. The configuration is illustrated in Figure 4(a), where ρ denotes the angle of incidence and ϕ defines the orientation of the in-plane static electric field **E**_0_ with respect to the *xz*-plane of incidence. While our analysis focuses on wave propagation within the *xz*-plane, the general scenario can be recovered by varying the direction of the in-plane bias **E**_0_. The TDBG is positioned at *z* = 0, sandwiched between two dielectric media with equal permittivity ϵ_1_ = ϵ_2_ = ϵ = 1. The optical fields **E**_ω_ and **B**_ω_ are assumed to have a positive wave vector component along the + *z*-direction, within the *xz* incidence plane. Our approach follows the scattering formalism introduced in ref,^41^ with a detailed derivation provided in the Supporting Information.^27,42,43^ The total effective conductivity, **σ**_eff_(ω), in the $E_{\omega }=E_{\omega }^{x}\mathrm{x̂}+E_{\omega }^{y}ŷ$ basis is

6 $$\sigma_{e\mathrm{ff}}=\frac{\sigma_{E}}{\gamma -i\omega }\left[\begin{array}{ll} 1 & 0\\ 0 & 1 \end{array}\right]+\frac{i\omega \alpha }{\gamma -i\omega }\left(\frac{\gamma \epsilon }{c}\right)\sin\rho \left[\begin{array}{ll} 0 & \sin\phi \\ 0 & -\cos\phi \end{array}\right]$$

where *c* denotes the speed of light, and γ = 10^11^ rad ·s^–1^ ≈ 0.02 THz represents the adopted scattering rate. We restrict the frequency of the incident wave to the range 0.02 THz ≪ ω ≪ 0.9 THz, with the lower bound ensuring validity within the dilute impurity limit, and the upper bound avoiding the onset of the interband transition regime. The latter is defined by the smallest optical gap ϵ_gap_ = 3.7 meV ≈0.9 THz, corresponding to a twist angle θ = 1.75° in TDBG under a vertical bias of *U* = 27.5 meV. The effective conductivity parameters, σ_*E*_ and α, were obtained from the BM model as outlined previously. Following refs 44,45 we define the ellipticity as ψ ≈ 32.982 × (*A*_*L*_ – *A*_*R*_), where *A*_*L*_ and *A*_*R*_ denotes the (dimensionless) absorbance of left-circularly polarized (LCP) and right-circularly polarized (RCP) light, respectively. The numerical prefactor of 32.982 converts the final result to degrees.^44,45^ In this work, we present our results in millidegrees, following the conventions of refs. 12,13,46

**Figure 4:**
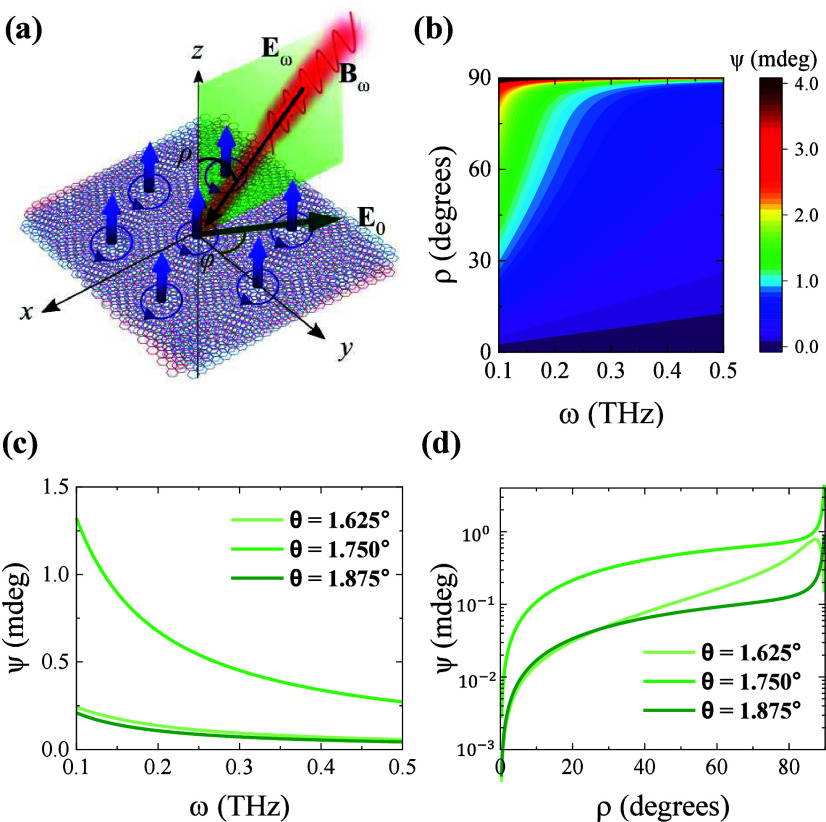
(a) Schematic of the system with the twisted double bilayer graphene at *z* = 0, in between dielectrics with relative permittivities ϵ_1_ (*z* < 0) and ϵ_2_ (*z* > 0). The incidence plane is depicted, and the incidence angle is ρ. Ellipticity (b) with respect to frequency and incidence angle ρ at θ = 1.750° twisting; (c) with respect to frequency at ρ = 45°; and (d) with respect to ρ at ω = 0.3 THz (see Figure 3 for corresponding σ_E_ and α). In panels (b)–(d), γ = 10^11^ rad·s^–1^, ϵ_1_ = ϵ_2_ = 1, *E*_0_ = 10^5^ V·m^–1^, ϕ = 90°.

The circular dichroism is caused by the imaginary component of the off-diagonal conductivity, that is Im(σeffxy) in eq 6, which induces a current in the + *x*-direction (−*x*) in response to RCP (LCP) light. In this work, we assume ϕ = 90° in order to maximize $\sigma_{e\mathrm{ff}}^{xy}$ . Figure 4(b) shows that the dichroism decreases with frequency but increases with incidence angle. The former case can be understood by noting that Im(σeffxy)→0 in the large frequency limit ω → *∞*. This behavior is more clearly illustrated in Figure 4(c), which also compares the results for TDBG twisted at θ = 1.625°, θ = 1.750°, and θ = 1.875°, all evaluated at a fixed incidence angle of ρ = 45°. Notably, the ellipticities obtained here are comparable to the THz circular dichroism observed in twisted bilayer graphene in ref,^12^ as well as to the pronounced dichroism reported in the visible to near-ultraviolet range in multilayer twisted graphene systems,^13^ both measured at normal incidence. In contrast to our study, these effects are attributed to in-plane magnetic moments^13^ and do not originate from a static field **E**_0_.

The enhancement of circular dichroism with increasing incidence angle is explicitly shown in Figure 4(d), and is now discussed in greater detail. This behavior originates from the Zeeman coupling between $B_{\omega }^{z}$ and $m_{nk}^{z}$ , which increases with ρ in our setup, as can be seen in eq 6. Note that this coupling causes the circular dichroism to vanish at normal incidence, since $B_{\omega }^{z}=0$ when ρ = 0. This feature offers a unique signature of optical dichroism induced by magnetoelectric EO effects, in striking contrast to previous studies.^12,13^ We also find that at large angles of incidence, the magnitude of the Drude conductivity plays a role in determining the circular dichroism spectra. For TDBG at θ = 1.625° a broader and smaller peak is observed in the spectra, which is due to the large Drude conductivity compared to the θ = 1.750° and θ = 1.875° cases (See Figure 3). The higher dissipative Drude component decreases the peak and the higher reactive component broadens it.^27^

We have shown how magnetoelectric EO effects manifest in metallic 2D systems, taking twisted double-bilayer graphene as a representative platform. While in realistic samples, strain-induced contributions originating from the GME and BCD may also be present and potentially comparable, the analysis presented here based on idealized *C*_3*z*_-symmetric systems reveals that the magnetoelectric EO gives rise to giant ellipticities displaying a unique dependence on the incidence angle, a feature that can be used to disentangle the effect from other possible coexisting contributions. The magnitude of the effect was found to be substantial, as concluded from two central observations: 1) The predicted ellipticities exceeding 1 mdeg in the THz regime require a substantial strength of the controlling effect in atomically thin films, since the “interaction volume” is substantially suppressed in these systems. 2) The magnetoelectric coefficients predicted in this work are remarkably large compared to the dynamical coefficients of other known systems, such as twisted bilayer graphene^40^ or even topological chiral crystals,^47^ under appropriate assumptions. To the best of our knowledge, α_max_ = 20000 μ_*B*_/V·nm is beyond the largest magnetoelectric coefficient reported in literature, indicating substantial bias-induced gyrotropy is possible in these systems.

## Supplementary Material



## Notes

Symmetry analysis of graphene-based moiré systems; details of the Bistritzer-MacDonald continuum model for TDBG; derivation of the effective gapped Dirac Hamiltonian for magnetoelectric response; and comprehensive scattering formalism for calculating optical dichroism in 2D systems (PDF)
