# Supplementary material for: Metallic Electrooptic Effect in Twisted Double-Bilayer Graphene
Source: Nano Lett. 2026 May 28;26(22):7235–41. doi: 10.1021/acs.nanolett.5c05662 (PMC13267174; doi:10.1021/acs.nanolett.5c05662)
Supplement: Supplementary file 1 [file nl5c05662_si_001.pdf]

Supplementary Information

Metallic Electro-Optic Effect in Twisted Double-Bilayer Graphene

D. J. P. de Sousa,[1] N. R.-Levchenko,[2,*] C. O. Ascencio,[2,*] J. D. S. Forte,[1] Paul M. Haney,[3] and Tony Low[1,4,†]

[1] *Department of Electrical and Computer Engineering, University of Minnesota, Minneapolis, Minnesota 55455, USA*
[2] *School of Physics and Astronomy, University of Minnesota, Minneapolis, Minnesota 55455, USA*
[3] *Physical Measurement Laboratory, National Institute of Standards and Technology, Gaithersburg, Maryland 20899-6202, USA*
[4] *Department of Physics, University of Minnesota, Minneapolis, Minnesota 55455, USA*

(Dated: May 19, 2026)

S1. VANISHING OF THE BERRY CURVATURE DIPOLE

In this section we demonstrate that the vanishing of the Berry curvature dipole in monolayer and (double) bilayer graphene systems is a consequence of the symmetry elements of their crystallographic point groups. The Fermi surface formula for the Berry curvature dipole is

$$D^{\alpha\beta} = \hbar \sum_{n\vec{k}} \left(-\frac{\partial f^0_{n\vec{k}}}{\partial \epsilon_{n\vec{k}}}\right) v^{\beta}_{n\vec{k}} \Omega^{\alpha}_{n\vec{k}} \tag{S1}$$

where we have included the factor of $\hbar$ for consistency with most literature. The presence of a symmetry, S, necessarily commutes with the Hamiltonian, which implies that $\epsilon_{n\vec{k}} = \epsilon_{n\vec{k'}}$, where $\vec{k'} = R_S\vec{k}$. Here R_S is an orthonormal matrix representation of the symmetry S, which acts on the crystal momenta $\vec{k}$. It follows that the energy derivative of the Fermi function must satisfy $\frac{\partial f^0_{n\vec{k}}}{\partial \epsilon_{n\vec{k}}} = \frac{\partial f^0_{n\vec{k'}}}{\partial \epsilon_{n\vec{k'}}}$. We note that the arguments presented here apply equally to the gyrotropic magnetic tensor ($\mathbf{K}$) on account of $\mathbf{K}$ being symmetry-equivalent to $\mathbf{D}$.

S1.A. Monolayer Graphene

The crystal lattice of monolayer graphene is generated by point group C_{6v} [S1]. The selected non-trivial point group generators consist of C_{2z}, a mirror line along the armchair direction, M_y, and C_{3z}, where z is the out-of-plane direction. Bloch electrons are confined to the xy-plane by virtue of the two-dimensional nature of the crystal, so the only possible non-zero components of the Berry curvature dipole are D^{zx} and D^{zy}. With respect to $\Omega^z_{n\vec{k}}$ and $\vec{v}_{n\vec{k}}$, C_{2z} acts as spatial inversion, i.e. $\Omega^z_{n\vec{k}} \to \Omega^z_{n(-\vec{k})}$ and $(v^x_{n\vec{k}}, v^y_{n\vec{k}}) \to (-v^x_{n(-\vec{k})}, -v^y_{n(-\vec{k})})$. Time-reversal symmetry imposes $\Omega^z_{n\vec{k}} = -\Omega^z_{n(-\vec{k})}$, so the coexistence of C_{2z} and time-reversal symmetries forces $\Omega^z_{n\vec{k}} = 0$. Hence, the concurrent presence of these two symmetries precludes the existence of the Berry curvature dipole via the vanishing of the Berry curvature throughout the Brillouin zone.

It is well known that a nonzero Berry curvature requires the breaking of time-reversal or spatial inversion symmetry. However, the presence of C_{2z} symmetry alone in two dimensional systems is sufficient for a vanishing Berry curvature dipole. The C_{2z} symmetry requires

$D^{z\beta} = \hbar \sum_{n\vec{k}} (-\frac{\partial f^0_{n\vec{k}}}{\partial \epsilon_{n\vec{k}}}) v^{\beta}_{n\vec{k}} \Omega^z_{n\vec{k}} = \hbar \sum_{n\vec{k}} (-\frac{\partial f^0_{n(-\vec{k})}}{\partial \epsilon_{n(-\vec{k})}})(-v^{\beta}_{n(-\vec{k})}))\Omega^z_{n(-\vec{k})} = -D^{z\beta}$,

where $\beta = x, y$. Therefore $D^{z\beta} = 0$.

The mirror symmetry, M_y, imposes the following constraints on the Berry curvature and velocity: $\Omega^z_{n\vec{k}} = -\Omega^z_{n\vec{k'}}$ and $(v^x_{n\vec{k}}, v^y_{n\vec{k}}) = (v^x_{n\vec{k'}}, -v^y_{n\vec{k'}})$, where $\vec{k'} = (k_x, -k_y)$. Substituting these constraints into eq. $S1$ and integrating over the first Brillouin zone reveals that $D^{zx} = -D^{zx} \implies D^{zx} = 0$, while D^{zy} can be nonzero as a consequence of the evenness of $v^y_{n\vec{k}}\Omega^z_{n\vec{k}}$ under the mirror operation. Thus, in the presence of a single mirror, a nonzero Berry curvature dipole will be directed perpendicular to it. In this specific case, it would point along the zig-zag direction.

Under a C_{3z} transformation $\Omega^z_{n\vec{k}} \to \Omega^z_{n\vec{k'}}$ and $(v^x_{n\vec{k}}, v^y_{n\vec{k}}) \to (-\frac{1}{2}v^x_{n\vec{k'}} - \frac{\sqrt{3}}{2}v^y_{n\vec{k'}}, \frac{\sqrt{3}}{2}v^x_{n\vec{k'}} - \frac{1}{2}v^y_{n\vec{k'}})$, where $\vec{k'}$ are the rotated crystal momenta. Inserting these into eq. $S1$ and noting that the threefold rotation maps $\vec{k}$ onto an equivalent point in reciprocal space reveals that $D^{zx} = -\frac{1}{2}D^{zx} - \frac{\sqrt{3}}{2}D^{zy}$ and $D^{zy} = \frac{\sqrt{3}}{2}D^{zx} - \frac{1}{2}D^{zy}$. These two expressions indicate that $D^{zx} = -\frac{\sqrt{3}}{3}D^{zy}$ and $D^{zy} = \frac{\sqrt{3}}{3}D^{zx} \implies D^{zx} = D^{zy} = 0$. Thus, the presence of C_{3z} alone is a sufficient condition for a vanishing Berry curvature dipole.

S1.B. AA-Stacked Bilayer Graphene

AA-stacked bilayer graphene is associated with point group D_{6h} [S2], which is generated by C_{3z}, C_{2z}, C_{2y}, and spatial inversion. Following the arguments of the previous section, C_{2z} and C_{3z} enforce the vanishing of $D^{z\beta}$, $\beta =$

* These authors contributed equally to this work.
† tlow@umn.edu

x, y. C_{2y} transforms the Berry curvature and velocity components according to $\Omega^z_{n\vec{k}} \to -\Omega^z_{n\vec{k}'}$, $v^x_{n\vec{k}} \to -v^x_{n\vec{k}'}$, and $v^y_{n\vec{k}} \to v^y_{n\vec{k}'}$, where $\vec{k}' = (-k_x, k_y, -k_z)$. Integrating over the first Brillouin zone gives $D^{zx} \neq 0$ and $D^{zy} = 0$. Spatial inversion acts in an identical manner to C_{2z} with respect to $\Omega^z_{n\vec{k}}$ and $v^{x,y}_{n\vec{k}}$, so $D^{z\beta}$ must vanish under spatial inversion symmetry. It is generally true that every component of the Berry curvature dipole must vanish when spatial inversion symmetry is present. This follows from the odd nature of polar vectors and the even nature of pseudovectors under spatial inversion (see eq. $S1$ and Monolayer Graphene section).

S1.C. AB Stacked Bilayer Graphene

Changing the stacking from AA type to AB(Bernal) type lowers the point group symmetry from D_{6h} to D_{3d} [S2, S3]. The generators of D_{3d} are C_{3z}, C_{2y}, and spatial inversion. Again, C_{3z} and spatial inversion symmetries enforce $D^{z\beta} = 0$, whereas only D^{zx} is symmetry-allowed by C_{2y}.

S1.D. Twisted Bilayer Graphene

Adding a twist to bilayer graphene reduces the point group symmetries from the untwisted cases. The possible set of point groups of twisted bilayer graphene, which depend on the stacking and twist angle, are D_6, D_3, C_6, or C_3 [S4]. The list of potential generators are C_{3z}, C_{2z}, and C_{2y}. As was already argued in previous sections, C_{3z} and C_{2z} enforce the vanishing of $D^{z(x,y)}$ (see section on Monolayer Graphene), whereas C_{2y} enforces the vanishing of D^{zy}, but not D^{zx}.

S1.E. Twisted Double Bilayer Graphene

The point group corresponding to the twisted double bilayer graphene system considered in this work (AB-AB stacked) is D_3 [S5]. Our specific choice of generators are C_{3z} and C_{2y}. As demonstrated in previous sections, these symmetries constrain the Berry curvature dipole components in the following way: $D^{z(x,y)} = 0$ w.r.t. C_{3z} and $D^{zx} \neq 0$, $D^{zy} = 0$ w.r.t C_{2y}.

It is important to note that each of the systems considered here has as one of its point group generators C_{3z}, a threefold axis orthogonal to the graphene plane for the monolayer case or in the stacking direction in the layered cases. As demonstrated through symmetry arguments, this symmetry necessitates that D^{zx} and D^{zy} vanish. Thus, the experimental observation of the effects of the Berry curvature dipole in these systems requires the breaking of this threefold rotational symmetry in addition to breaking C_{2z} or spatial inversion when they are present and is usually accomplished by the application of strain.

S2. ELECTRONIC STRUCTURE OF TWISTED-DOUBLE BILAYER GRAPHENE

Our calculations use the BM model for twisted double bilayer graphene [?]. The Hamiltonian in the $\boldsymbol{K}$-valley, written in the spinor basis $\psi = (\phi, \chi)$—where ϕ and χ denote the respective four-component spinors for the top and bottom bilayers—can be expressed as

$$\mathcal{H}_{\text{TDBG}} = \mathcal{H}_{\text{BLG}}(\theta)\left(I + \frac{\tau_3}{2}\right) + \mathcal{H}_{\text{BLG}}(-\theta)\left(I - \frac{\tau_3}{2}\right) + \mathcal{T}(\mathbf{r})\tau^+ + \mathcal{T}^\dagger(\mathbf{r})\tau^-, \tag{S2}$$

where τ_i are Pauli matrices in the layer degree of freedom and $\tau^\pm = (\tau_x \pm i\tau_y)/2$. The bilayer graphene Hamiltonian $\mathcal{H}_{\text{BLG}}$ is given by

$$\mathcal{H}_{\text{BLG}} = \begin{pmatrix} U_1 + \Delta & -\gamma_0 f(\mathbf{k}) & \gamma_4 f^*(\mathbf{k}) & \gamma_1 \\ -\gamma_0 f^*(\mathbf{k}) & U_1 & \gamma_3 f(\mathbf{k}) & \gamma_4 f^*(\mathbf{k}) \\ \gamma_4 f(\mathbf{k}) & \gamma_3 f^*(\mathbf{k}) & U_2 & -\gamma_0 f(\mathbf{k}) \\ \gamma_1 & \gamma_4 f(\mathbf{k}) & -\gamma_0 f^*(\mathbf{k}) & U_2 + \Delta \end{pmatrix}, \tag{S3}$$

where $f(\mathbf{K} + \mathbf{k}) = \frac{3}{2}(-k_x + ik_y)a_0$ and $a_0 = 0.246\,\text{nm}$ is the graphene lattice constant. We adopt the zero-angle approximation $\mathcal{H}_{\text{BLG}}(\theta) \to \mathcal{H}_{\text{BLG}}$, valid for small twist angles.

The interlayer tunneling between bilayers is captured by the tunneling matrix

$$\mathcal{T}(\mathbf{r}) = \begin{pmatrix} 0 & 0 \\ T(\mathbf{r}) & 0 \end{pmatrix}, \tag{S4}$$

where the tunneling function is given in momentum space by $T(\mathbf{r}) = \sum_{n=1}^{3} T_n e^{i\mathbf{q}_n \cdot \mathbf{r}}$. The T_n matrices are expressed as

$$T_n = w_{AB} \left(\eta I + \cos[(n-1)\phi_0]\sigma_x + \sin[(n-1)\phi_0]\sigma_y \right), \tag{S5}$$

with $\phi_0 = 2\pi/3$, $\eta = w_{AA}/w_{AB}$, and σ_i denoting Pauli matrices in the sublattice basis. The tunneling matrices are related by C_{3z} symmetry of the moiré lattice via the unitary transformation $U_{3z}(\phi_0) = \exp(i\sigma_z\phi_0/2)$.

The momentum transfer vectors corresponding to the moiré reciprocal lattice are:

$$\mathbf{q}_1 = k_\theta(0, -1), \tag{S6}$$

$$\mathbf{q}_2 = k_\theta \left(\frac{\sqrt{3}}{2}, \frac{1}{2} \right), \tag{S7}$$

$$\mathbf{q}_3 = k_\theta \left(\frac{\sqrt{3}}{2}, -\frac{1}{2} \right), \tag{S8}$$

where $k_\theta = \frac{4\pi}{3a_M}$ and $a_M = \frac{a_0}{2\sin(\theta/2)}$ is the moiré lattice constant.

The Hamiltonian in the $\boldsymbol{K'}$-valley is obtained via time-reversal symmetry from the $\boldsymbol{K}$-valley Hamiltonian.

We use the following parameters in our simulations (in meV):

$$(\gamma_0/a_0, \gamma_1, \gamma_3/a_0, \gamma_4/a_0, \Delta) = (2338, 361, 245, 119, 15).$$

The interlayer tunneling amplitudes are

$$(w_{AA}, w_{AB}) = (88, 110)\,\text{meV},$$

where w_{AA} and w_{AB} denote tunneling amplitudes between AA and AB stacked sublattices, respectively. The electrostatic potential due to a vertical displacement field is modeled as a linear potential drop of magnitude U.

The out-of-plane component of the momentum-resolved orbital magnetization for a given band n is given by:

$$m^z_{n\mathbf{k}} = e\hbar \sum_{m \neq n} \frac{\mathrm{Im}[\langle n|v^x_{n\mathbf{k}}|m\rangle\langle m|v^y_{n\mathbf{k}}|n\rangle]}{E_{n\mathbf{k}} - E_{m\mathbf{k}}} \tag{S9}$$

where $f_{n\mathbf{k}} = [1 + \exp[(E_{n\mathbf{k}} - \mu)/k_B T]]^{-1}$ is the Fermi-Dirac distribution at temperature T and chemical potential μ, and $v^{x,y}_{n\mathbf{k}} = (1/\hbar)\partial H/\partial k_{x,y}$ are velocity operators. The Berry curvature $\Omega_n(\mathbf{k})$ of the nth band is defined in a similar manner as

$$\Omega^z_{n\mathbf{k}} = -2\hbar^2 \sum_{m \neq n} \frac{\mathrm{Im}[\langle n|v^x_{n\mathbf{k}}|m\rangle\langle m|v^y_{n\mathbf{k}}|n\rangle]}{(E_{n\mathbf{k}} - E_{m\mathbf{k}})^2}. \tag{S10}$$

The electro-optic tensor of interest is then obtained by multiplying these quantities at every $\mathbf{k}$ point within the moiré Brillouin zone.

S3. SCREENING EFFECT ON THE ELECTRONIC STRUCTURE OF TWISTED DOUBLE BILAYER GRAPHENE

We have investigated the effects of electronic screening on the band structure of twisted double-bilayer graphene (TDBLG) by incorporating Hartree potentials following the self-consistent approach described in Ref. [S6]. At the twist angles considered here, the low-energy bands are neither flat nor quasi-flat, suggesting that screening-induced band renormalization should be minimal. To verify this, we compared single-particle band structures (solid black curves) with those renormalized by the Hartree potential (dashed red curves) under various vertical biases and filling factors ν for a single-valley, spinless electron model.

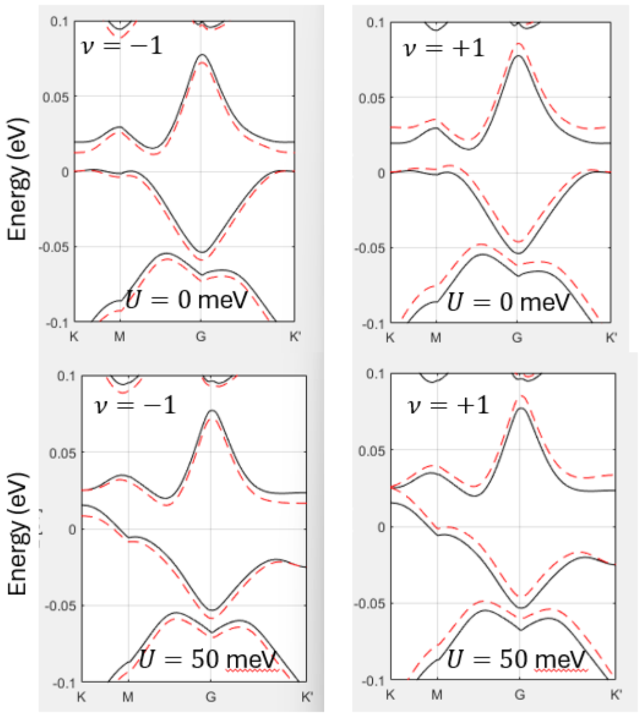

FIG. S1. Ellipticity at large incidence angles and varying Drude conductivities. The solid (dashed) line corresponds to $\mathrm{Re}(\sigma_{\mathrm{Drude}}) = \sigma_0^{\mathrm{Re}}$ $(= 100\sigma_0^{\mathrm{Re}})$. From light green to dark green, $\mathrm{Im}(\sigma_{\mathrm{Drude}}) = \sigma_0^{\mathrm{Im}},\ 10\sigma_0^{\mathrm{Im}},\ 100\sigma_0^{\mathrm{Im}}$. Here, $\chi^{zz} = G(\theta = 1.750°)$, $f = 0.3$ THz, $\gamma = 10^{11}$ rad·s^{-1}, $\epsilon_1 = \epsilon_2 = 1$, $E_0 = 10^5$ V·m^{-1}, $\phi = 90°$.

Our results indicate that the Hartree potential does not qualitatively alter the electronic spectrum at these angles. Although quantitative modifications to the energy gap occur, where the gap may increase or decrease with the filling factor at a fixed bias, the underlying topological and symmetry features remain robust. Because the predicted maximization of the magnetoelectric electro-optic response near gap-closing transitions depends primarily on these qualitative band features, giant response peaks are expected to persist even in the presence of screening. The primary effect of screening is a renormalization of the external bias U_{ext} into an effective bias $U_{\mathrm{eff}} = U_{\mathrm{ext}} + U_{\mathrm{ind}}(\nu)$, where the induced potential U_{ind} is determined through a self-consistent analysis.

S4. BIAS INDUCED CONDUCTIVITY FOR THE GAPPED DIRAC MODEL

Here, we derive practical analytical expressions for the various conductivity coefficients in a 2D gapped Dirac electron system. These results, primarily used as references in the main text, are presented explicitly here for completeness. The Hamiltonian is $H_\eta = \hbar v_F(\eta\sigma_x k_x + \sigma_y k_y) + (\Delta/2)\sigma_z$, where $\eta = \pm 1$ is the valley index, v_F is the fermi velocity, Δ quantifies the size of the energy gap and σ_j, $j = x, y, z$, are the Pauli matrices. The relevant quantities, such as orbital moments and berry curvature have already been derived somewhere else [S7]

S4.A. Definitions

The orbital magnetization is defined as

$$m_\eta^z(\mathbf{k}) = \frac{\eta e}{4\hbar}(\hbar v_F)^2 \frac{\Delta}{\left[\left(\frac{\Delta}{2}\right)^2 + (\hbar v_F k)^2\right]}, \tag{S11}$$

and the Berry curvature is given by

$$\Omega^z_\eta(\mathbf{k}) = \frac{\eta}{4}(\hbar v_F)^2 \frac{\Delta}{\left[\left(\frac{\Delta}{2}\right)^2 + (\hbar v_F k)^2\right]^{1/2}}, \tag{S12}$$

where $\eta = \pm 1$, depending on the valley. The conductivities $\sigma_E(\omega)$ and $\sigma_B(\omega)$ are equal to

$$\sigma_E(\omega) = \frac{e^2\tau}{1-\omega\tau}\int \frac{d^2k}{(2\pi)^2}\left[-\frac{\partial f(\epsilon-\mu)}{\partial \epsilon_{\eta,k}}\right] v_{\eta,k} v_{\eta,k}, \tag{S13}$$

$$\sigma_B(\omega) = \frac{e^2\tau}{1-\omega\tau}\int \frac{d^2k}{(2\pi)^2}\left[-\frac{\partial f(\epsilon-\mu)}{\partial \epsilon_{\eta,k}}\right] m^z_\eta(\mathbf{k})\Omega^z_\eta(\mathbf{k}), \tag{S14}$$

where $v_{\eta,k} = 1/\hbar \partial\epsilon_\eta(\mathbf{k})/\partial k$ is the velocity operator. The monolayer graphene bands are given by

$$\epsilon_\eta(\mathbf{k}) = \pm\sqrt{\left(\frac{\Delta}{2}\right)^2 + (\hbar v_F k)^2}, \tag{S15}$$

so the velocity operator is thus

$$v_\eta(k) = \pm\left[\left(\frac{\Delta}{2}\right)^2 + (\hbar v_F k)^2\right]^{-1/2} \hbar^2 v_F^2 k. \tag{S16}$$

S4.B. Calculation of $\sigma_E(\omega)$

First, we start changing the integration from $k-$space to integrating over the energies ϵ. From Eq. S15, we obtain that

$$k = \pm\frac{1}{\hbar v_F}\sqrt{\epsilon^2 - \left(\frac{\Delta}{2}\right)^2}, \tag{S17}$$

from which,

$$dk = \frac{1}{\hbar v_F}\frac{\epsilon}{\left[\epsilon^2 - \left(\frac{\Delta}{2}\right)^2\right]^{1/2}} d\epsilon. \tag{S18}$$

and

$$kdk = \frac{1}{(\hbar v_F)^2}\epsilon d\epsilon. \tag{S19}$$

Therefore,

$$\int F(k)\frac{d^2k}{(2\pi)^2} = \frac{1}{(2\pi)^2}\int_0^{2\pi}\int_0^\infty F(k) k dk d\phi = \frac{1}{2\pi}\int_0^\infty \frac{\epsilon}{(\hbar v_F)^2} F(\epsilon) d\epsilon. \tag{S20}$$

Using Eqs. S17 and S16,

$$v_\eta(\epsilon) = \frac{v_F}{\epsilon}\sqrt{\epsilon^2 - \left(\frac{\Delta}{2}\right)^2}, \tag{S21}$$

where we have assumed $d^2k = kdkd\phi$. Plugging this result into S14,

$$\begin{aligned}
\sigma_E &\propto \frac{1}{2\pi}\int_0^\infty \left[-\frac{\partial f(\epsilon-\mu)}{\partial\epsilon}\right] v^2(k)kdk \\
&\sim \frac{1}{2\pi}\int_0^\infty \delta(\epsilon-\mu)\frac{v_F^2}{\epsilon^2}\left[\epsilon^2-\left(\frac{\Delta}{2}\right)^2\right]\frac{1}{(\hbar v_F)^2}\epsilon d\epsilon \\
&\sim \frac{1}{2\pi\hbar^2}\int_0^\infty \delta(\epsilon-\mu)\left[\epsilon^2-\left(\frac{\Delta}{2}\right)^2\right]\frac{d\epsilon}{\epsilon} \\
&\sim \frac{1}{2\pi\hbar^2}\left[\mu^2-\left(\frac{\Delta}{2}\right)^2\right]\frac{1}{\mu} \\
&\sim \frac{\mu}{2\pi\hbar^2}\left[1-\left(\frac{\Delta}{2\mu}\right)^2\right].
\end{aligned} \tag{S22}$$

where we have assumed that the derivative of the Fermi-Dirac distribution with respect to the energy is approximately a Dirac delta function in the limit $T \to 0$. Including the pre-factor $\tau/(1-i\omega\tau)$,

$$\sigma_E(\omega) = \frac{e^2}{1-i\omega\tau}\frac{\mu}{2\pi\hbar^2}\left[1-\left(\frac{\Delta}{2\mu}\right)^2\right]. \tag{S23}$$

S5. CALCULATION OF $\sigma_B(\omega)$

Following along the same lines, we start by changing Eqs.S11 and S12 from momentum to energy:

$$\Omega_\eta^z(\mathbf{k}) = \frac{\eta}{4}(\hbar v_F)^2\frac{\Delta}{\epsilon^3} \tag{S24}$$

$$m_\eta^z(\mathbf{k}) = \frac{\eta e}{4\hbar}(\hbar v_F)^2\frac{\Delta}{\epsilon^2}. \tag{S25}$$

Hence,

$$\begin{aligned}
\sigma_B(\omega) &\propto \int \frac{d^2k}{(2\pi)^2}\left[-\frac{\partial f(\epsilon-\mu)}{\partial\epsilon}\right]\Omega_\eta^z(\mathbf{k})m_\eta^z(\mathbf{k}) \\
&\sim \frac{1}{2\pi}\int_0^\infty \delta(\epsilon-\mu)\frac{(\hbar v_F)^2}{16\hbar}\frac{\Delta^2}{\epsilon^5}\epsilon d\epsilon \\
&\sim \frac{e(\hbar v_F)^2\Delta^2}{32\pi\hbar\mu^4}.
\end{aligned} \tag{S26}$$

And finally,

$$\sigma_B(\omega) = \frac{i\omega\tau}{i\omega\tau-1}\frac{e(\hbar v_F)^2\Delta^2}{32\pi\hbar\mu^4}. \tag{S27}$$

S6. DICHROISM

S6.A. Scattering Formalism

Without loss of generality, we assume that the wave is incident in the xz-plane and traveling in the +z direction. For clarity purposes, the phase factors are omitted. Thus, the incident, reflected, and transmitted components of the E and H fields may be written as [?]:

$$E_{inc} = \begin{bmatrix} -a_p\cos\rho_1 \\ a_s \\ a_p\sin\rho_1 \end{bmatrix} \quad H_{inc} = \frac{n_1}{z_0}\begin{bmatrix} -a_s\cos\rho_1 \\ -a_p \\ a_s\sin\rho_1 \end{bmatrix} \tag{S28}$$

$$E_{ref} = \begin{bmatrix} r_p \cos\rho_1 \\ r_s \\ r_p \sin\rho_1 \end{bmatrix} \quad H_{ref} = \frac{n_1}{z_0} \begin{bmatrix} r_s \cos\rho_1 \\ -r_p \\ r_s \sin\rho_1 \end{bmatrix} \tag{S29}$$

$$E_{tr} = \begin{bmatrix} -t_p \cos\rho_2 \\ t_s \\ t_p \sin\rho_2 \end{bmatrix} \quad H_{tr} = \frac{n_2}{z_0} \begin{bmatrix} -t_s \cos\rho_2 \\ -t_p \\ t_s \sin\rho_2 \end{bmatrix} \tag{S30}$$

where the subscripts s and p denote the s-polarized and p-polarized components of the wave, respectively; ρ_1 and ρ_2 are the angles of incidence and transmission, respectively; and z_0 is the vacuum impedance. Lastly, ρ_1 is related to ρ_2 by Snell's law, i.e. $n_1 \sin\rho_1 = n_2 \sin\rho_2$.

The boundary conditions which relate the fields on the top and bottom dielectrics are:

$$E_{tr}^{x,y} = E_{inv}^{x,y} + E_{ref}^{x,y} \tag{S31}$$

$$H_{tr}^{x} - H_{inc}^{x} - H_{ref}^{x} = \sigma_{yx}(\omega) E_{tr}^{x} + \sigma_{yy}(\omega) E_{tr}^{y} \tag{S32}$$

$$H_{tr}^{y} - H_{inc}^{y} - H_{ref}^{y} = -\sigma_{xx}(\omega) E_{tr}^{x} - \sigma_{xy}(\omega) E_{tr}^{y} \tag{S33}$$

Combining in Eqs. S28-S30 into Eqs. S31-S33 and solving for t_s,t_p,r_s,r_p in terms of a_s, a_p, we get:

$$\mathbf{t_s} = \frac{2n_1 \alpha \cos\rho_1}{\lambda} \mathbf{a_s} + \frac{2n_1 \kappa \cos\rho_1}{\lambda} \mathbf{a_p} \tag{S34}$$

$$\mathbf{t_p} = \frac{2n_1 \eta \cos\rho_1}{\lambda} \mathbf{a_s} + \frac{2n_1 \beta \cos\rho_1}{\lambda} \mathbf{a_p} \tag{S35}$$

$$\mathbf{r_s} = \left(\frac{2n_1 \alpha \cos\rho_1}{\lambda} - 1 \right) \mathbf{a_s} + \frac{2n_1 \kappa \cos\rho_1}{\lambda} \mathbf{a_p} \tag{S36}$$

$$\mathbf{r_p} = -\frac{2n_1 \eta \cos\rho_2}{\lambda} \mathbf{a_s} + \left(1 - \frac{2n_1 \beta \cos\rho_2}{\lambda} \right) \mathbf{a_p} \tag{S37}$$

where $\alpha = n_1 \cos\rho_2 + n_2 \cos\rho_1 + z_0 \sigma_{xx} \cos\rho_2 \cos\rho_1$,
$\eta = z_0 \sigma_{xy} \cos\rho_2$, $\kappa = z_0 \sigma_{yx} \cos\rho_1$, $\beta = n_1 \cos\rho_1 + n_2 \cos\rho_2 + z_0 \sigma_{yy}$, and $\lambda = \alpha\beta - \eta\kappa$. The handedness-dependent absorption is given by

$$A_{R,L} = I - T_{R,L} - R_{R,L}, \tag{S38}$$

where

$$T_{R,L} = \frac{\epsilon_2 \cos\rho_2}{2\epsilon_1 \cos\rho_1} \begin{bmatrix} 1 & \pm i \end{bmatrix} \begin{bmatrix} t_{11} & t_{21}^* \\ t_{12}^* & t_{22} \end{bmatrix} \begin{bmatrix} t_{11} & t_{12} \\ t_{21} & t_{22} \end{bmatrix} \begin{bmatrix} 1 \\ \mp i \end{bmatrix}, \tag{S39}$$

$$R_{R,L} = \frac{1}{2} \begin{bmatrix} 1 & \pm i \end{bmatrix} \begin{bmatrix} r_{11} & r_{21}^* \\ r_{12}^* & r_{22} \end{bmatrix} \begin{bmatrix} r_{11} & r_{12} \\ r_{21} & r_{22} \end{bmatrix} \begin{bmatrix} 1 \\ \mp i \end{bmatrix}. \tag{S40}$$

S6.B. Conductivity manipulations

The current response to oblique-incident light may be summarized as

$$\mathbf{J} = \boldsymbol{\sigma}_{\text{Drude}}\mathbf{E}_\omega + \boldsymbol{\sigma}_{\text{G}}\mathbf{B}_\omega. \tag{S41}$$

For the system studied in this paper, it is possible to write the B-field contribution to the current response in terms of the transverse E-field components. The precise form of $\boldsymbol{\sigma}_{\text{G}}$ is given in the maintext as

$$\boldsymbol{\sigma}_{\text{G}} = \frac{e^2}{\hbar}\frac{i\omega\tau}{i\omega\tau - 1}\mathbf{F}_0 \cdot \mathbf{G}, \tag{S42}$$

where, assuming a 2D system and an in-plane static bias with magnitude E_0 traveling in the $(\cos\phi, \sin\phi)$ direction,

$$\mathbf{F}_0 = E_0 \begin{bmatrix} 0 & 0 & \sin\phi \\ 0 & 0 & -\cos\phi \\ -\sin\phi & \cos\phi & 0 \end{bmatrix}, \mathbf{G} = \begin{bmatrix} 0 & 0 & 0 \\ 0 & 0 & 0 \\ 0 & 0 & \chi^{zz} \end{bmatrix} \to \sigma_B \propto E_0\chi^{zz} \begin{bmatrix} 0 & 0 & \sin\phi \\ 0 & 0 & -\cos\phi \\ 0 & 0 & 0 \end{bmatrix} \tag{S43}$$

The $\mathbf{G}$ tensor is what couples the static bias, herein represented by the $\mathbf{F}_0$ tensor, to the AC current response. Thus, it is evident that the component of the current which couples to the B-field is only dependent on the B-field's longitudinal component (ie, z-component). From boundary conditions for a 2D interface, this component is conserved [S8].

For a monochromatic incident wave, such as the one assumed in this paper, the B-field may be related to the E-field as such [S9]

$$\begin{bmatrix} B^x \\ B^y \\ B^z \end{bmatrix} = \frac{\sqrt{\epsilon_{r,i}}}{c} \begin{bmatrix} 0 & -\hat{k}_{z,i} & \hat{k}_{y,i} \\ \hat{k}_{z,i} & 0 & -\hat{k}_{x,i} \\ -\hat{k}_{y,i} & \hat{k}_{x,i} & 0 \end{bmatrix} \begin{bmatrix} E^x \\ E^y \\ E^z \end{bmatrix}, \tag{S44}$$

where $\hat{k}_{n,m}$ are the nth component of the $\hat{k}$ unit vector in the ith medium. B^z is then equivalent to $-\hat{k}_y E^x + \hat{k}_x E^y$. Note that by assuming propagation in the xz-plane, $\hat{k}_{y,i} = 0 \Rightarrow \hat{k}_{x,i} = \sin\rho_i$. Since the quantity $\sqrt{\epsilon_r}\sin\rho$ is conserved by Snell's law, it suffices to only consider $\sqrt{\epsilon_{r,2}}\sin\rho_2$. Hence, rewriting Eq. S42 in the E-field basis by using Equation S44 and combining the expression for B-field coupling with the known E-field coupling component allows us to re-write the conductivity in terms of the E-field. Namely,

$$\boldsymbol{\sigma}_{\text{eff}} = \frac{e^2}{\hbar}\frac{\gamma/\pi}{\gamma - i\omega}\begin{bmatrix} \sigma_{\text{E}} & 0 \\ 0 & \sigma_{\text{E}} \end{bmatrix} + \frac{e^2}{\hbar}\frac{i\omega\sqrt{\epsilon_2}\sin(\rho_2)}{\gamma - i\omega}\left(\frac{E_0\chi^{zz}}{c}\right)\begin{bmatrix} 0 & \sin(\phi) \\ 0 & -\cos(\phi) \end{bmatrix}. \tag{S45}$$

In the main text, the subscript below ϵ and ρ is omitted, since $\epsilon = \epsilon_1 = \epsilon_2$ is assumed. We emphasize that the components of the matrix to the right of the addition symbol (referred to as $\boldsymbol{\sigma}_{\text{G}}^{\text{NH}}$ from here) in Eq. S45 depend on the incidence plane of $\mathbf{E}_\omega$. If the optical field were incident in the yz-plane instead of the xz-plane, we would have $\sigma_{\text{G}}^{\text{NH};xx}, \sigma_{\text{G}}^{\text{NH};yx} \neq 0$ and $\sigma_{\text{G}}^{\text{NH};xy}, \sigma_{\text{G}}^{\text{NH};yy} = 0$, which is consistent with $\mathbf{G}$ only coupling with B^z.

S6.C. Drude response

In the following section, we summarize the effects of the Drude conductity on the ellipticity spectra at large incidence angles. The baseline Drude conductivity used in the following section corresponds to $\chi^{zz} = G(\theta = 1.750°)$, which in numerical terms is

$$\sigma_0 = \frac{e^2}{\hbar}\frac{\gamma/\pi}{\gamma - i\omega}\sigma_{\text{E}} \approx (0.49 + 9.22i) \cdot 10^{-6}\Omega^{-1}, \tag{S46}$$

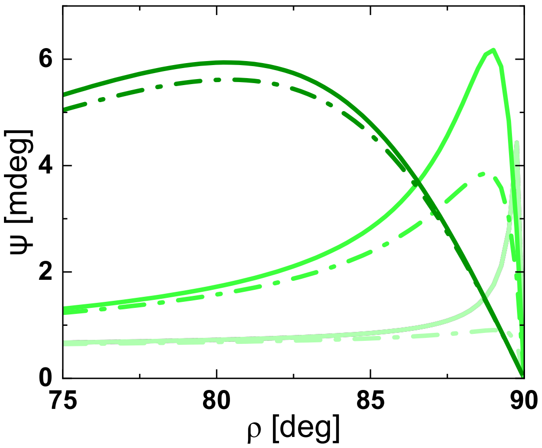

FIG. S2. Ellipticity at large incidence angles and varying Drude conductivities. The solid (dashed) line corresponds to $\mathrm{Re}(\sigma_{\mathrm{Drude}}) = \sigma_0^{\mathrm{Re}}$ $(= 100\sigma_0^{\mathrm{Re}})$. From light green to dark green, $\mathrm{Im}(\sigma_{\mathrm{Drude}}) = \sigma_0^{\mathrm{Im}}$, $10\sigma_0^{\mathrm{Im}}$, $100\sigma_0^{\mathrm{Im}}$. Here, $\chi^{zz} = G(\theta = 1.750°)$, $f = 0.3$ THz, $\gamma = 10^{11}$ rad·s^{-1}, $\epsilon_1 = \epsilon_2 = 1$, $E_0 = 10^5$ V·m^{-1}, $\phi = 90°$.

assuming that $\gamma = 10^{11}$ rad·s^{-1} and $f = 0.3$ THz. Let the real and imaginary components of the expression in Eq. S46 be labeled σ_0^{Re} and σ_0^{Im} respectively.

[S1] R. Battilomo, N. Scopigno, and C. Ortix, Phys. Rev. Lett. **123**, 196403 (2019), URL `https://link.aps.org/doi/10.1103/PhysRevLett.123.196403`.

[S2] W.-Y. He, D. Goldhaber-Gordon, and K. T. Law, Nature Communications **11**, 1650 (2020), ISSN 2041-1723, URL `https://doi.org/10.1038/s41467-020-15473-9`.

[S3] E. McCann and M. Koshino, Reports on Progress in Physics **76**, 056503 (2013), URL `https://dx.doi.org/10.1088/0034-4885/76/5/056503`.

[S4] L. Zou, H. C. Po, A. Vishwanath, and T. Senthil, Phys. Rev. B **98**, 085435 (2018), URL `https://link.aps.org/doi/10.1103/PhysRevB.98.085435`.

[S5] M. Koshino, Phys. Rev. B **99**, 235406 (2019), URL `https://link.aps.org/doi/10.1103/PhysRevB.99.235406`.

[S6] T. Cea, N. R. Walet, and F. Guinea, Phys. Rev. B **100**, 205113 (2019), URL `https://link.aps.org/doi/10.1103/PhysRevB.100.205113`.

[S7] D. Xiao, W. Yao, and Q. Niu, Phys. Rev. Lett. **99**, 236809 (2007), URL `https://link.aps.org/doi/10.1103/PhysRevLett.99.236809`.

[S8] P. A. D. Goncalves and N. M. R. Peres, An Introduction to Grpahene Plasmonics (World Scientific Publishing, 2016), chap. 2, p. 14, 1st ed.

[S9] D. J. Griffiths, Introduction to Electrodynamics (Reed College, 2017), chap. 9, pp. 405–406, 4th ed.
